# I Don’t See It Your Way: The Dot Perspective Task Does Not Gauge Spontaneous Perspective Taking

**DOI:** 10.3390/vision2010006

**Published:** 2018-02-08

**Authors:** Stephen R. H. Langton

**Affiliations:** Faculty of Natural Sciences, University of Stirling, Stirling FK9 4LA, UK; stephen.langton@stir.ac.uk; Tel.: +44-1786-467-659

**Keywords:** perspective taking, social attention, vision, theory of mind, gaze-cued attention

## Abstract

Data from studies employing the dot-perspective task have been used to support the theory that humans are capable of automatically computing the visual perspective of other individuals. Recent work has challenged this interpretation, claiming instead that the results may arise through the automatic reorienting of attention triggered by observed head and gaze cues. The two experiments reported here offer a stronger test of the perspective taking account by replacing the computer-generated avatars used in previous research with, respectively, photo-realistic stimuli and socially co-present individuals in a “live”, face-to-face version of the task. In each study observers were faster to judge the number of dots in a display when either a digitized image depicting a human “gazer” (Experiment 1), or a socially co-present gazer (Experiment 2) could see the same number of dots as the observer, than when the number of dots visible to each was different. However, in both experiments this effect was also obtained in conditions where barriers clearly occluded the gazers’ view of the target dots so that the perspectives of participants and gazers were always different. These results offer no support for the idea that participants are engaged in spontaneous perspective taking in the dot perspective task. It is argued that, instead, the results are likely caused by a spontaneous redirection of a viewer’s attention by the observed gazes, which is unlikely to involve representations of the gazer’s mental state.

## 1. Introduction

Consider the following scenario. You are eating breakfast with a friend. She glances toward a plate of pancakes and says: “would you like another?” It will be readily apparent to you or to any observer within earshot that your friend is enquiring about your desire or otherwise for another pancake, rather than, say, another cup of coffee, marriage, child, or back operation. We are clearly able to track one another’s gazes, compute their perspectives and then use this information to make inferences about uttered remarks. Indeed, effective social interaction and communication in general demands that we impute perceptions and beliefs to other individuals based on their observable behaviour. Recent work by Apperly and colleagues [1,2,3,4] has suggested that this is achieved through the operation of two distinct processing systems. The first is cognitively demanding, relatively slow, flexible, and linked to executive functioning and language abilities. It allows us to reason about the full range of propositional attitudes such as perceptions, beliefs, and desires. The second system is fast, automatic, and inflexible and uses a less sophisticated conceptual repertoire to *track* others’ propositional attitudes rather than actually represent them as such; this implicit mentalizing system encodes such relations between agents and objects as “encountering” and “registering”, which are supposed to be relationships that serve as proxies for, respectively, perceiving and believing. It has been argued that this system enables us to take on the visual perspective of another individual automatically and spontaneously [4,5,6,7], although the evidence for this has been disputed [7,8,9,10,11]. This controversy is the topic of the present article.

The evidence in support of spontaneous visual perspective taking comes from the dot perspective task developed by Samson and colleagues [4]. In their original study participants were presented with a computer-generated human avatar standing in the centre of a virtual room facing either the left or right hand wall. A number of discs were presented on one or both walls such that on some trials the participant was able to see the same number of discs as the avatar (the consistent condition), while on other trials the avatar could only see some of the discs (the inconsistent condition). For example, on an inconsistent trial the avatar might be facing the right wall on which there are two discs, while a single disc is positioned on the left-hand wall behind her; the avatar can therefore see two discs while the participant can see all three discs by virtue of the fact that he or she can see both walls of the room. On a consistent trial, on the other hand, the avatar might be facing the left-hand wall on which there are three discs, while there are no discs on the right-hand wall; both avatar and participant can therefore see three discs. On each trial before the presentation of the virtual room display, participants were first presented with a perspective instruction (e.g., the words “You” or “Her”) followed by a digit display (a “1”, “2” or “3”). Under the “You” perspective instruction, the participant’s task was to judge whether or not the number of discs visible in the room matched the number given in the digit display (i.e., a self-perspective judgement). Under the “She” instruction, the task was to judge whether or not the number of discs seen by the avatar matched the number in the digit display (i.e., an other-perspective judgement). The key finding for the current purpose was that the time taken by participants to judge the number of discs that they themselves could see was influenced by the number of discs that were visible to the avatar. More specifically, reaction time (RT) was faster on consistent trials, when both participant and avatar could see the same number of discs, then on inconsistent trials, where the number of discs visible to each was different. Moreover, this consistency effect was obtained even in an experiment where participants were only ever asked to make the judgements from their own perspective, or when participants were simultaneously engaged in a demanding secondary task [5]. Samson et al.’s [4] interpretation of these data is that participants automatically compute the avatar’s perspective, which, on inconsistent trials, somehow interferes with the processing of their own perspective, slowing their response time relative to consistent trials where computations of self- and other-perspectives yield the same result.

The spontaneous perspective taking interpretation has its detractors, however [8,9,10,11,12]. For example, Santiesteban et al. [9] used the dot perspective task but also included a condition where an arrow replaced the avatar. Both types of directional cue yielded consistency effects of comparable magnitude leading the authors to suggest that the result is due to domain-general processing, rather than the automatic tracking of mental states by observers. Wilson and colleagues reached the same conclusion having obtained equivalent consistency effects with an avatar, an arrow and a camera [12]. Specifically the suggestion is that the consistency effect could be explained by the automatic orienting of participants’ attention triggered by the directional information provided by avatars and arrows [2,3,13,14,15]. For example, on consistent trials participants’ attention will be oriented toward the side of the display containing all of the discs, which may facilitate the computation of the number of visible discs, relative to the inconsistent condition where attention will be drawn to the side of the room containing either no discs or a merely subset of the total number of discs.

As recently pointed out by Cole et al. [8], however, the observation that non-social or obviously mindless directional stimuli like arrows or cameras can generate comparable consistency effects to avatars is not sufficient to falsify the spontaneous perspective taking hypothesis. For example, it may be that the consistency effect can be generated by either a directional cueing effect, or the automatic generation of another’s perspective, or potentially both. Indeed, Nielson et al. [6] also obtained reliable consistency effects using avatars and arrows but also with dual-coloured blocks. Unlike Santiesteban et al. [9], however, they found a positive linear relationship between the magnitude of the consistency effect and the degree of social relevance of the stimuli: the socially relevant avatars produced the largest effect, non-social dual-coloured blocks the smallest effect and arrows—regarded as “semisocial”—produced an intermediate effect. Plausibly, these results might reflect domain-general visual orienting of attention operating in conjunction with spontaneous perspective taking, with the latter serving to generate larger consistency effects with social and semi-social stimuli. Then again, the same pattern of data could be generated if social stimuli like the avatars were more potent at triggering shifts of visual attention than arrows, or the asymmetric block stimuli.

Rather than seeking to demonstrate that non-social stimuli can also generate consistency effects, another strategy has been to test whether or not the effect is eliminated under conditions where perspective taking should not be possible. For example, Wilson et al. [12] reported an experiment using the normal avatar-in-a-room scenario but included a condition where the avatar was wearing a blindfold. Equivalent consistency effects were obtained in both cases, arguing against the perspective taking account. One problem with this method, however, is that the critical manipulation designed to eliminate the possibility of perspective taking is confounded with a change in the perceptual features of the avatar stimulus. Furlanetto et al. [7] overcame this problem by manipulating participants’ beliefs about identical avatars across different conditions. Participants were presented with the usual avatar-in-a-room stimuli on some trials but on others the avatar was wearing one of two different coloured goggles. Participants were led to believe that goggles of one colour were opaque, while those of the other colour were transparent. Normal consistency effects were obtained both when the avatar’s eyes were visible and when participants believed it was wearing transparent goggles but the effect was eliminated in trials where participants believed that the goggles were opaque, as is predicted by the perspective taking account of the consistency effect.

More recently, however, Conway et al. [10] argued that the Furlanetto et al. [7] result was likely to have been caused by the fact that participants were asked to make both self- and other-perspective judgements within each block of trials. More precisely, through the intermixing of self- and other-perspective trials, Furlanetto et al.’s participants may have been induced into making explicit judgements about the avatar’s perspective even when this was not required. This makes it impossible to determine whether the opaque goggles manipulation was exerting an effect on an explicit mentalizing process, or on the putative automatic perspective taking mechanism. Using a design in which participants made only self-perspective judgements, Conway et al. [10] failed to replicate the Furlanetto et al. [7] result. Instead, consistency effects were obtained under conditions where participants did and did not believe that the avatar could see the target dots, providing no support for the spontaneous perspective taking hypothesis.

A final strategy has been to use barriers in order to disrupt the line of regard from the avatar to the target objects. This removes the need for the avatar to wear odd-looking goggles or blindfolds but also has the advantage of keeping the gazing stimuli (i.e., the avatars) identical across the critical conditions. For example, Baker and colleagues [16] placed their avatar on the far left or right of a virtual room but such that it always faced into the room’s centre where the target stimuli were located. An occluding barrier was used to block the avatar’s line of sight to one, two or all three targets arranged either horizontally or vertically in the centre of the room. In this way, the participant was able to see all the targets, while the barrier was used to introduce the perspective inconsistency. Baker et al. obtained the usual consistency effect using this arrangement but also showed that its magnitude was reduced when gaps were placed in the barriers so that the previously occluded targets became visible to the avatar.

On the face of it, this result supports the perspective taking hypothesis; however, as with Furlanetto et al.’s opaque goggles experiment [7], Baker et al. intermixed self- and other-perspective trials, which, as already mentioned, may encourage participants to make explicit judgements about the avatar’s perspective even when this was not required. This potential limitation is not apparent in Cole et al.’s recent study [8]. Cole et al. employed the more typical stimuli with the avatar placed in the centre of a virtual room and used the presence or absence of an occluding barrier as a means of manipulating conditions under which perspective taking should or should not occur. The usual consistency effect was observed in a “seeing” condition, where avatars were clearly able to see the dots; however, they found an equivalent effect in the “non-seeing” condition where the imposition of the barrier meant that the avatar’s perspective differed from the participants on every trial, a finding which is not predicted by the perspective-taking hypothesis.

On balance then, the results discussed above do not lend much support to the notion that humans spontaneously compute others’ perspectives. Yet this conclusion may be too hasty, for in all of the studies described, as well as in all the other published work using the dot perspective task, computer generated avatars were used as the stimuli whose perspectives participants were supposed to compute. While it is clear that such stimuli can generate visual orienting effects [1], it is not obvious that they are ideal stimuli for the automatic attribution of visual perspective. Following Wiese and colleagues [17] it is suggested that participants must first adopt an intentional stance toward an agent before implicit mentalizing processes can come online. That is, one must perceive an agent as, for example, having a mind or being capable of referring to things outside of itself, before one is able to represent its behaviour in terms of mental states like “perceiving,” “attending” or “believing,” or even to track such states using relations like “encountering” or “registering.” In support of this view, a recent study by Gardner et al. [18] indicated that the consistency effect in the dot-perspective task did not occur when participants were unaware that they were involved in a perspective taking experiment. One interpretation of this result is that in the absence of instructions about self- and other-perspectives, participants did not adopt the intentional stance toward the avatar that may be necessary for implicit mentalizing to occur.

Findings from other related areas of research also lend support to this position. This work shows that a range of effects in visual attention are different when real people are used instead of schematic stimuli [19]. The phenomenon of “social” Inhibition of Return (IOR) is a good case in point. Inhibition of return is the slowing of responses to stimuli at recently attended locations [20]. This phenomenon is not readily observed when the stimulus directing attention is a central cue such as an arrow and only obtained with central gaze cues at relatively long cue-to-target intervals [21]; however, robust effects are found if the central cue is a real individual who interacts with the participant [22]. Other studies have suggested that the gaze-cued orienting effect is larger with a real, co-present gazer, as opposed to schematic gaze cues [23] and that participants are less likely to attend to the face of a real, co-present actor in a room, than to the same actor’s face on a computer monitor placed in the same room [24]. One reason for these differences may well be the increased likelihood of endoding a real, co-present agent’s gazes in terms of his or her mental states, than those of a schematic face, or even a photograph of a real face.

It is therefore possible that the use of computer-generated avatars in the dot perspective task does not provide a sufficiently stringent test of the spontaneous perspective taking hypothesis; participants may not have taken an intentional stance toward these stimuli, so that the consistency effects that have been observed are more likely to be the result of, for example, involuntary orienting of attention, which can be triggered by directional stimuli including schematic faces and arrows. Accordingly, in the present studies we adopted Cole et al.’s [8] occluding barrier technique but used photo-realistic stimuli in place of avatars in Experiment 1 and a novel “live,” face-to-face version of the task in Experiment 2.

If spontaneous perspective taking is at the root of the consistency effect found by Samson et al. [4], then it should be obtained in the seeing conditions of Experiments 1 and 2 but it should be absent, or at least greatly reduced, in the non-seeing conditions. Should the consistency effect be un-modulated by the imposition of the barrier, this would suggest an underlying cause other than spontaneous perspective taking. 

## 2. Experiment 1

In this experiment the computer generated avatar-in-a-room stimuli used in previous studies were replaced by photo-realistic stimuli. Digitised colour photographic images were obtained of a person sitting in the corner of a room whose head could be turned to face one or other of the walls. (Hereafter, what is termed a “gaze” actually comprises a combined head orientation and eye direction cue.) The targets on each trial were between one and three large blue discs mounted on these walls. On each trial, participants were presented with a digit in the centre of the screen. They were then shown the display of the person sitting in the room and they were asked to judge whether or not the number of dots visible in the room was the same as the number depicted in the digit display. On matching trials, the number shown at the start of the sequence corresponded to the number of dots in the room; on non-matching trials, the number in the digit display differed from the number of dots in the room. Unlike Samson et al.’s [4] study but in line with the studies reported by Cole et al. [8] and Conway et al. [10], each participant was only ever asked to make the matching/non-matching judgement from their own perspective. In some trials the person in the room (the gazer) had a clear view of the target dots (the “seeing” condition), while in others, barriers were positioned between the model and the targets (the “non-seeing” condition). One other difference between Experiment 1 and Cole et al.’s [8] occluding barrier study was that in Experiment 1 the seeing and non-seeing trials were selected randomly within each block of trials, whereas trials were blocked by visibility by Cole et al. [8]. Finally, as in Samson et al.’s [4] original study the main manipulation was of the consistency between the number of dots visible to the participant and the number of dots faced by the person in the display: on consistent trials they were the same, whereas on inconsistent trials they were different.

### 2.1. Experiment 1 Method

#### 2.1.1. Participants

Twenty-four undergraduates at the University of Stirling were recruited through opportunity sampling to take part in this experiment (Mean age = 23.5 years, *SD* = 6.8 years, range = 18–44 years). There were 18 females and six males all with normal or corrected-to-normal vision. Written informed consent was obtained from all participants. Ethical approval was granted by the University’s Ethics Committee.

#### 2.1.2. Materials and Apparatus

**Figure 1:**
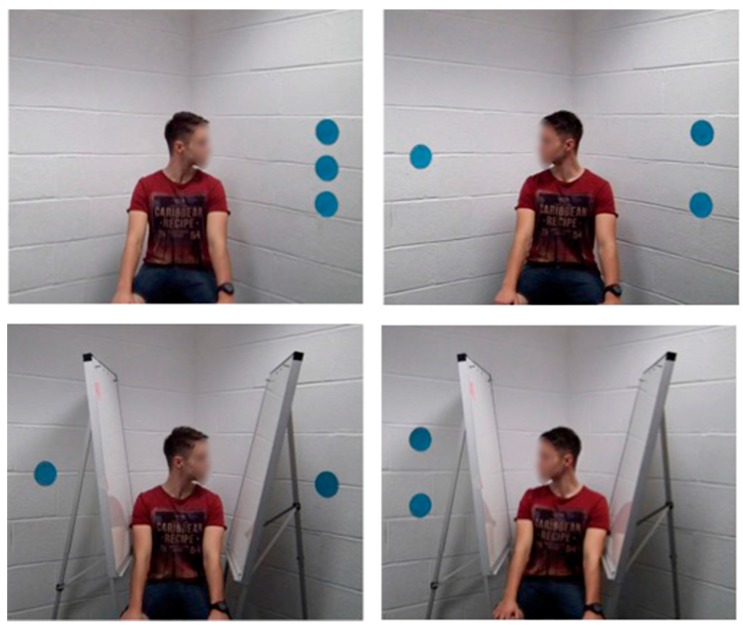
Examples of the stimuli used in Experiment 1. The top row depicts images in the seeing condition with different arrangements of three target discs. The bottom row contains examples of stimuli in the non-seeing condition with different arrangements of two target discs. The face of the gazer was not blurred in the actual experiment.

The stimuli for the experiment were digital colour images of a male individual (the gazer) sitting in the corner of a white-walled room with his head turned to either the left or right at an angle of approximately 90 degrees from straight ahead. Separate images were prepared with between one and three round blue paper discs affixed to the wall approximately 50 cm from the gazer’s head. These discs could appear on either the left or right of the gazer’s head and, where two or more discs appeared on one side, these were arranged vertically (see Figure 1). Separate images were prepared with all possible arrangements of the discs on either side of the gazer’s head. For example, for stimuli containing two discs, one image contained two discs on the left wall and none on the right, one contained two discs on the right and none on the left and another had one disc on either side. One set of these images was prepared with the gazer’s head oriented to the left and a second set with his head oriented to the right. This resulted in a total of 18 different images. A second set of images were prepared in the same way but with a pair of barriers interposed between the gazer and the target discs (see Figure 1). When viewed on the screen, the images all measured 10 cm wide by 8 cm in height. The target discs were 0.8 cm in diameter and aligned 4 cm to the left or right from the centre of the screen. In images where three discs appeared on the same side, the distance between from the top of one disc and the disc above was 0.2 cm and the distance from the bottom of the array of discs to the top was 3 cm. Where two discs appeared on the same side, these occupied the top and bottom positions as in the three-disc array. The separation from top of one disc to the bottom of the other was 1.2 cm.

The stimuli were viewed on a 19-inch monitor driven by a Lenovo personal computer from a distance of approximately 60 cm. E-Prime software (Psychology Software Tools, Pittsburgh, PA, USA) was used to present the stimuli. Responses were collected using a Serial Response Box (Psychology Software Tools, Pittsburgh, PA, USA).

#### 2.1.3. Design

The materials were tested in a factorial design with two within-subjects variables: consistency–whether the number of discs faced by the gazer and visible to the participant were the same (consistent) or different (inconsistent)–and visibility, which manipulated whether or not the model was able to see the target discs displayed on the walls (seeing versus non-seeing). Reaction time (RT) was recorded as the dependent variable.

#### 2.1.4. Procedure

Each trial began with a 750 ms presentation of a fixation cross in the centre of the screen. The screen then went blank and remained so for a further 500 ms before the word “You” was presented in the centre of the screen. This prompt remained on the screen for 750 ms after which the screen went blank. After a delay of 500 ms, the digit cue (1, 2, or 3) appeared on the screen for 750 ms. This was then replaced by the target image, which remained on the screen until the participant responded. Participants were asked to press the rightmost button on the response box if the digit cue matched the total number of discs seen in the target image, or the leftmost key if the digit and number of discs were mismatched. They were also asked to ignore the person sitting in the centre of the room and were given no other information about the gazer’s role. Reaction time was recorded from the onset of the target image until the execution of the participant’s response.

There was a total of 192 experimental trials comprising 24 trials in each of 8 cells made up of the factorial combination of trial type (matching versus non-matching trials), consistency (consistent versus inconsistent trials) and visibility (seeing versus non-seeing trials). Of these 24 trials, eight contained one disc, eight contained two discs and eight contained three discs. In half of the consistent trials, the target discs were presented on the left-hand side of the target image and half on the right-hand side. For those inconsistent trials where two discs appeared, there were an equal number with two discs on the left-hand side of the display, two on the right-hand side and one on each side. Where three discs appeared in inconsistent trials, there were similarly an equal number of trials with all possible arrangements of discs across the two sides of the display.

Trials were selected randomly from the complete set of 192 trials and arranged into four blocks of 48 trials. Participants were offered a break after each block. Participants also completed a set of 24 practice trials before the start of the first experimental block. These trials were also selected randomly from the complete pool of trial types so that participants would experience both seeing and non-seeing visibility conditions. After completing all of the experimental trials, participants were asked whether or not they believed that the barriers occluded the gazer’s view of the target discs. All agreed that they did.

#### 2.1.5. Data Analysis

In keeping with previous studies using the dot perspective task, reaction time data were only analysed from match trials-those whether the digit given at the start of the trial matched the number of dots seen by the participant in the subsequent display. As pointed out by Samson et al. [4] on mismatching trials the target digit denoted a number of dots seen by neither the participant nor the model and so these trials are likely to be much easier to process than matching trials. The median of correct RTs from match trials was computed for each participant in each condition and the data were entered into a 2 × 2 factorial (ANOVA) with consistency (consistent versus inconsistent) and visibility (seeing versus non-seeing) as within-subjects factors. Where a participant made errors on more than 40% of trials in any one condition, their data were excluded from further analysis.

### 2.2. Experiment 1 Results and Discussion

One participant’s data were excluded from further analysis because of an error rate exceeding 40% in one or more of the experimental conditions. For each of the remaining 23 participants, their median correct RTs were computed for each condition of the experiment.

**Figure 2:**
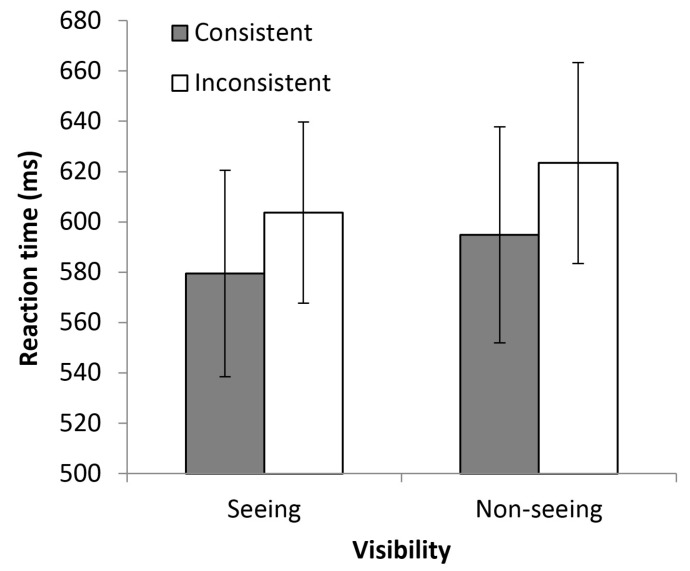
Mean RTs in each condition of Experiment 1. Error bars represent the standard error of the mean.

The inter-participant means of the median RTs on matching trials are summarised in Figure 2. As can be seen in this figure, participants’ RTs were faster on trials where the number of dots they could see was consistent rather than inconsistent with the number of dots faced by the model, regardless of whether the model had an unimpeded view of the dots (the seeing condition), or whether their line of regard was impeded by the barrier (the non-seeing condition). These observations are supported by the results of an analysis of variance (ANOVA) with consistency and visibility as repeated measures factors. This analysis yielded a main effect of consistency, *F*(1, 22) = 14.09, *p* = 0.001, η*_p_*^2^ = 0.39, with faster responding on consistent trials (*M* = 584 ms, *SE* = 39 ms) than on inconsistent trials (*M* = 610 ms, *SE* = 39 ms). There was no main effect of visibility (*p* = 0.22), and, critically, the interaction between consistency and visibility did not reach significance (*p* = 0.79).

Participants produced accurate responses on 95% of all trials and there was no evidence of a tradeoff between speed and accuracy that might compromise interpretation of the RT data (see Table 1). No further analyses were conducted on these data.

The aim of Experiment 1 was to replicate the consistency effect found by Samson et al. [4] and others using photo-realistic stimuli and to test whether or not the effect would be modulated by the introduction of a barrier that occluded the gazer’s view of the target discs. The results indicate that when the gazer’s view of the discs was unimpaired (the “seeing” condition), participants’ RTs were slower when the number of target discs in the gazer’s view was inconsistent with the number of discs faced by the participant, relative to situations where the number of discs faced by the gazer and seen by the participant were consistent. However, this consistency effect was uninfluenced by the imposition of the barrier, suggesting that, whatever underlies the effect, it is unlikely to be participants’ spontaneous adoption of the visual perspective of the gazer.

Obviously this conclusion rests on the effectiveness of the barrier in preventing the gazer from seeing the target discs. More precisely, it relies on participants perceiving that the gazer’s line of regard to the discs is impeded, which in turn should prevent them from spontaneously representing a relation between the gazer and the target objects. Although our manipulation check suggests that the barrier was effective in being perceived as blocking the gazer’s view, it is possible that during the actual experiment the intermixing of seeing and non-seeing trials within a block may have undermined the effectiveness of the barrier as a means of preventing spontaneous perspective taking. For example, the relation between the gazer and the target discs that may have been spontaneously established in a seeing trial early in a block of trials may nevertheless persist in subsequent non-seeing trials that, after all, involve the same agent in the same context, save for the presence of the occluding barriers. A better test of the spontaneous perspective-taking hypothesis would therefore involve the blocking of trials by the visibility manipulation. This issue is addressed in Experiment 2.

Experiment 1 used photo-realistic stimuli in order to encourage participants to adopt an intentional stance toward the gazer. This, it was argued, is a necessary precursor to automatic mentalizing, including spontaneous perspective taking. Despite this, we found no evidence that the consistency effect was modulated by the visibility or otherwise of the targets (notwithstanding the issue of intermixing seen and unseen trials discussed above). It is of course possible that participants failed to adopt an intentional stance even under these conditions. In order to address this issue, Experiment 2 involved a “live” actor as the gazer who sat face-to-face with the participant. Under these circumstances, it would presumably be very hard for participants not to regard the gazer as an intentional agent. This arrangement should therefore provide a stronger test of the spontaneous-perspective taking hypothesis.

## 3. Experiment 2

Experiment 2 used a modified version of the dot-perspective task with a live actor replacing the photographic stimuli used in Experiment 1. Participants sat face-to-face with the gazer and the target dots appeared on computer monitors positioned on either side of the gazer, such that they were in full view of both individuals in the “seeing” condition. In the “non-seeing” conditions barriers were placed between the gazer and the monitors so that the targets could be seen by the participant but not by the gazer. On each trial, the gazer was cued to look toward one or other monitor shortly before the target dots appeared. As in Experiment 1, participants were asked to make a judgement about whether or not the number of dots matched a number that they were given at the start of the trial. The key manipulation was once again the consistency, or otherwise, of the perspectives of the gazer and participant; on consistent trials, each could see the same number of dots, whereas on inconsistent trials the number of target dots faced by the gazer differed from the number visible to the participant. Reaction times were expected to be faster in the former than the latter in the seeing condition; however, under the perspective-taking hypothesis, this consistency effect was expected to be reduced or absent in the non-seeing condition.

Rather than intermixing seeing and non-seeing trials within a block, trials in Experiment 2 were blocked by visibility with block order counterbalanced between participants. This arrangement allowed us to examine whether the order in which seen and unseen trials are encountered can influence the magnitude of the consistency effect.

### 3.1. Experiment 2 Method

#### 3.1.1. Participants

Thirty-six people participated in Experiment 2 (mean age = 25 years; *SD* = 9.3 years; age range = 17–56, 21 females and 15 males). All were gathered through opportunity sampling and had normal or corrected-to-normal vision and hearing. Ethical approval for the study was granted by the University Ethics Committee.

#### 3.1.2. Materials and Apparatus

**Figure 3:**
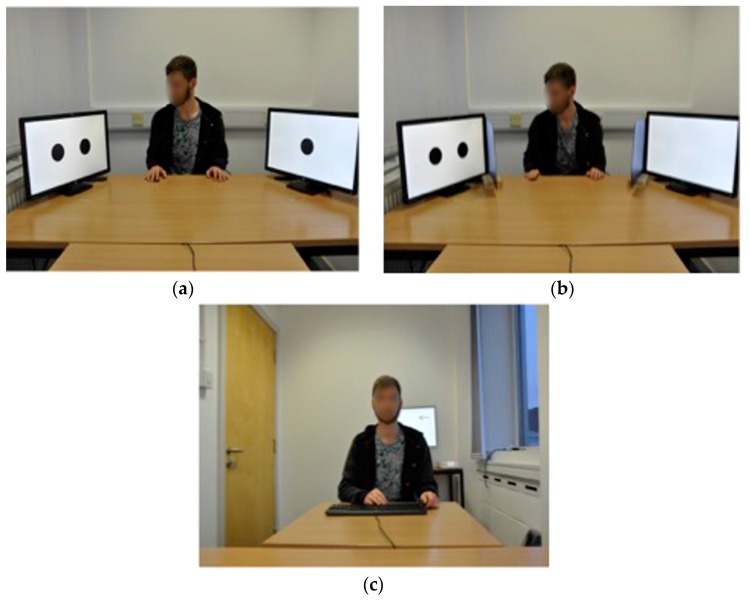
The arrangement of the apparatus used in Experiment 2: (**a**) from the point of view of a participant in the “seeing” condition; (**b**) from the point of view of a participant in the “non-seeing” condition; (**c**) from the point of view of the gazer where an arrow cue instructing the gazer to direct his gaze to the left can be seen above the participant’s left shoulder.

The experiment took place in a rectangular room, with a window covered by blinds on one side. Two rectangular tables were arranged in a “T” shape in the centre of the room, with the top of the T at the narrow end of the room (see Figure 3). The length of the two tables combined in this shape was 173 cm. The experimenter, who acted as the gazer in this experiment, sat in the centre of the wide end of the T shape and the participant sat at the thin end. Three computer monitors were used in total. Two identical 24 inch HP Elite Display E231 monitors were placed to the left and right of the gazer and arranged at an angle that meant that the closest point of the monitor was 53 cm from the centre of the top of the table and the furthest point was 64 cm (see Figure 3). Each of these two monitors could display either one, two or three black discs arranged in a horizontal line. These discs were each 7.5 cm in diameter. In trials where three discs appeared on a single monitor the discs were separated by a 1 cm gap and in trials with two discs, the separation was 9.5 cm. The third monitor, a 19 inch HP Compaq LA1951g display, was placed behind the participant 250 cm from the end of the T where the gazer sat. This monitor could display an arrow pointing toward either the left or right, indicating the direction that the gazer should look on each trial (see Figure 3c).

In the barrier condition, two opaque, black barriers were placed long side down between the gazer and the monitors (see Figure 3b). The barriers were made of thin black plastic boards that measured 37 cm wide by 29 cm that sat in a thin slot cut into small wooden blocks; these blocks added an extra 4 cm to the total height of the barrier. Although slightly smaller than the surface area of the 24 inch screens these barriers were more than large enough to block the gazer’s view of the stimuli. An audio speaker was used to play pre-recorded numbers. A standard HP keyboard was used to record responses from participants and the experiment was run on an HP computer, linked to all three monitors and running E-Prime software (Psychology Software Tools, Pittsburgh, PA, USA).

#### 3.1.3. Design

The materials were tested in a mixed design with consistency and visibility as within-subjects variables and block order (seeing first versus non-seeing first) as a between-subjects variable. The dependent variable was reaction time.

#### 3.1.4. Procedure

The participant and experimenter sat facing each other at opposite ends of the table. At the start of each trial an electronic speaker positioned to the left of the participant played an audio clip of the word “one,” “two” or “three.” Following a delay of 500 ms, the experimenter was cued to turn his head toward one or other of the two adjacent monitors by the appearance of an arrow, which was displayed on the third monitor positioned behind the participant’s head. Two seconds after the arrow was displayed, the monitors adjacent to the experimenter displayed a total of one, two or three discs. Participants were asked to press the “m” key on the keyboard in front of them if the total number of discs displayed across both monitors matched the number played the audio clip at the start of the trial and the “z” key if the total number of discs did not match the number in the audio clip. The discs disappeared after the participant responded and the screens remained blank for 500 ms until the start of the next trial. Participants were told that the experimenter would turn to look at one of the monitors before the target discs were presented but they were not explicitly told to ignore the experimenter and nor were they given any information about any predictive relationship between the gaze direction and the correct response.

The total number and composition of the experimental trials were identical to those of Experiment 1 with the following exceptions. First participants were given 12 practice trials before the first block of experimental trials. For practical reasons these trials were of the same visibility condition as the participant’s first block of experimental trials, but they were randomly selected from within the total pool of possible trials for that particular visibility type. Second, before beginning the “non-seeing” experimental trials, all participants were explicitly asked whether or not they believed the barriers occluded the gazer’s view of the target dots. All participants indicated that the barriers were effective in this regard.

#### 3.1.5. Data Analysis

The treatment of the RT data was identical to that of Experiment 1; however, in Experiment 2 these data were analysed with a mixed factorial ANOVA with consistency (consistent versus inconsistent) and visibility (seeing versus non-seeing) as within-subjects factors and block order (seeing first versus non-seeing first) as a between subjects factor.

### 3.2. Experiment 2 Results and Discussion

**Figure 4:**
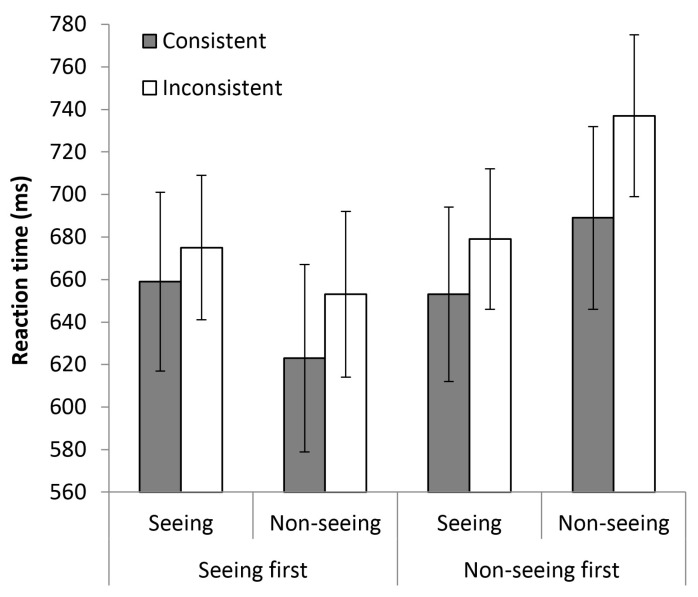
Mean RTs obtained in Experiment 2 as a function of visibility, consistency and block order. Error bars represent the standard error of the mean.

One participant’s error rate exceeded 40% in one or more conditions of the experiment and their data was therefore excluded from further analysis. The means of the median RTs of the remaining 35 participants are summarised in Figure 4. Regardless of whether the barrier was present or absent, participants were slower to respond on trials where the number of dots they could see in the display was inconsistent with the number of dots facing the model, relative to trials where the number of dots seen by the participant was consistent with that faced by the model. Moreover, there is no suggestion that the consistency effect is any larger on seeing versus non-seeing trials. Indeed, if anything the size of the effect is actually greater in the non-seeing condition for both block orders.

In support of these observations, there was a significant main effect of consistency, *F*(1, 33) = 5.31, *p* = 0.028, η*_p_*^2^ = 0.14, with faster responses on consistent trials (*M* = 656 ms, *SE* = 29 ms) than inconsistent trials (*M* = 686 ms, *SE* = 25 ms). The interaction between consistency and visibility failed to reach significance, *F*(1, 33) = 1.19, *p* = 0.28. There were no significant main effects of visibility or block order (*p*s > 0.48) and no evidence of interactions between consistency and block order, or between consistency, block order and visibility (*p*s > 0.6). The only other effect that reached significance was the interaction between visibility and block order, *F*(1, 33) = 6.29, *p* = 0.013, η*_p_*^2^ = 0.17. Those participants who completed the seeing blocks first were, on average, faster to respond on trials in the non-seeing blocks (*M* = 638 ms, *SE* = 40 ms) than the seeing blocks (*M* = 667 ms, *SE* = 36 ms), whereas those who completed the non-seeing blocks first showed the opposite pattern: faster responding on trials in the seeing blocks (*M* = 666 ms, *SE* = 35 ms) than the non-seeing blocks (*M* = 713 ms, *SE* = 39 ms). This is simply a practice effect: participants were, on average, faster to respond in blocks of trials they completed later rather than earlier in the experiment.

As in Experiment 1, participants performed at or near ceiling accuracy on the task, averaging 94% across all trials. There was no evidence suggestive of a systematic trade-off between speed and accuracy (see Table 1). No further analyses were conducted on these data.

Experiment 2 used a novel, live face-to-face version of the dot perspective task in order to provide a further test of the perspective-taking account of the consistency effect under conditions that were arguably ideal for participants to adopt an intentional stance toward the gazer. Once again, when the gazer had an unimpeded view of the target dots the consistency effect was obtained: participants were faster to respond on trials where the number of target dots visible to themselves was identical to the number of dots faced by the gazer, compared with trials where a different number of dots were faced by the gazer and visible to the participant. However, this consistency effect was also obtained when a barrier clearly occluded the gazer’s line of sight to the targets, a result that is not predicted by the spontaneous perspective-taking hypothesis. Indeed, the consistency effect was actually numerically larger, though non-significantly so, under non-seeing than seeing conditions. Moreover, the order in which the seeing and unseeing blocks were completed made no difference to the magnitude of the consistency effect. This result argues against any carry-over of spontaneous perspective-taking from a seeing block of trials to subsequent non-seeing blocks; the consistency effect occurred in the non-seeing condition even for participants who had yet to experience a single trial where the gazer could see the targets.

## 4. General Discussion

The experiments reported here were designed to test the hypothesis that the consistency effect, which has been repeatedly obtained in various versions of the dot perspective task, is underpinned by spontaneous perspective taking–a form of implicit mentalizing. These experiments sought to do so under conditions where participants would more readily adopt an intentional stance toward the gazing agent than in previous studies using the avatar-in-a-room stimuli and therefore provide a better test of the hypothesis. Consistency effects were readily obtained in Experiment 1, which used photorealistic stimuli and in Experiment 2, which used a novel live face-to-face version of the task. However, in neither experiment was there any evidence that the magnitude of the effect was influenced by the imposition of a barrier between the gazing agent and the target objects, a result that would be predicted by the spontaneous perspective taking account.

The results of Experiments 1 and 2 are in line with those of Cole et al. [8] who also failed to find evidence of a modulation of the consistency effect using the barrier manipulation, as well as Conway et al. [10] who, instead of using a physical barrier, manipulated participants’ beliefs about whether or not the gazing agent was able to see. These findings all run counter to the claim that the consistency effect in the dot perspective task gauges a form of implicit mentalizing.

A supporter of the implicit mentalizing explanation might argue that these manipulations do not provide adequate tests of the perspective-taking account. For example, it may be that there is no information flow between the implicit and explicit mentalizing systems, in which case there would be no reason to expect the implicit encoding of an agent’s perspective to be influenced by an explicit belief about their inability to see. On this line of argument, evidence from studies involving the manipulation of participants’ beliefs should certainly be discounted, regardless of whether the data support the perspective taking account [7] or not [10]. The barrier studies could be regarded in the same way: it could be argued that the imposition of the barrier serves to influence participants’ explicit beliefs about whether or not the gazing agent can see the target dots, but there is no reason to think that such beliefs should necessarily impact on the operation of the implicit system. This is misguided, however, for the idea is that the barrier serves to manipulate participants’ perception of the physical properties of the environment and, in particular, the physical relation between the gazer and that environment. According to the theory developed by Apperly and colleagues, this kind of information is available to the implicit mentalizing system; they are explicit that the “encountering” relation between an agent and an object-the representation that is supposed to track the agent’s perception and therefore their visual perspective-can only obtain if the object is not occluded [2,3]. So, although it is certainly possible that participants form explicit beliefs about what the gazing agent can or cannot see when a barrier is introduced, it is not these beliefs that are supposed to impact upon the implicit system; instead the barrier manipulates a relation that should be encoded by the implicit system itself and which should therefore have an impact on the consistency effect. The fact it does not is therefore problematic for the theory.

Rather than resulting from spontaneous perspective taking, it seems more likely that, as others have suggested [8,9], the consistency effect obtained in the dot perspective task is generated by the automatic shifting of observers’ visual attention triggered by the avatar’s gaze direction–so-called gaze-cued attention [13,25,26]. Samson et al. [4] argued that that this phenomenon was indeed likely to *contribute* to the consistency effect; however, the weight of evidence favours this explanation as its sole cause. The distinction between the perspective taking and gaze-cued orienting explanations of the consistency effect is important because only the former demands that the observer encodes a mental relation between the agent and the gazed-at target; gaze-cued orienting demands no such relation. Indeed, several authors have suggested that it is a rather primitive behavioural response that serves to orient an observer’s attention in the general direction in which another’s eyes and/or head have rotated [13,23,27]. The fact that gaze cued orienting is also unaffected by the same kind of barrier manipulation used in the present experiments lends further support for it being the mechanism responsible for the consistency effect [28].

One argument against this kind of gaze cueing explanation for the consistency effect arises from the results of a recent study by Gardner et al. [18]. They showed that the Samson et al. avatar-in-a-room stimuli were capable of triggering gaze-cued orienting effects but only at longer stimulus onset asynchronies (SOAs)–the time between the appearance of the gazing avatar and the to-be-detected target discs. The gaze-cueing effect was significant at an SOA of 600 ms, only marginally significant at an SOA of 300 ms and entirely absent with an SOA of 0 ms. The SOA between gazer and targets in Experiment 1 of the present paper was also 0 ms. The claim might therefore be that the stimuli used in this study are incapable of triggering the kind of attention shifts that are supposed to account for the consistency effect. However, it is worth pointing out that the cueing paradigm is partly designed to estimate the time course of the deployment of attentional resources in response to a cueing stimulus. The Gardner et al. result suggests that it takes at least 300 ms for the gaze cue to be encoded and attention to be oriented accordingly, which is in line with many other findings in the gaze-cueing literature. This is ample time for attention to influence responding in the dot perspective task where RTs are typically in the order of 600–700 ms. Second, the same criticism does not apply to Experiment 2, which involves a non-zero SOA. (The actual SOA will depend on the speed with which the gazer registers and responds to the arrow prompt, which will vary somewhat from trial-to-trial; however, a rough estimate would be between 1000–1500 ms.) Finally, at least one other study suggests that avatars *are* capable of triggering a gaze-cueing effect at 0 ms, as long as participants’ attention is initially directed at the avatar’s location [1]. Given these consideration, it is argued that gaze-cued attention remains plausible as a mechanism underpinning the consistency effects observed in the dot-perspective task.

Before proceeding, it is worth discussing a potential confound that, on the face of it, might undermine any conclusions about the extent to which the version of the dot-perspective task used here and elsewhere in the literature [4,7,8,12] has anything to do with the gaze direction of the person or avatar in the centre of the room. The arrangement of the stimuli in the two main categories of trials is such that, on consistent trials discs are always presented on one side of the gazer, while on many but not all, of the inconsistent trials, the discs are distributed between the two walls (e.g. the gazer faces two discs, while a third disc is on the wall behind him). The concern is therefore that the consistency effect arises from, say, slower processing of the discs when they are more widely separated regardless of whether participants processed the perspective of the gazer, simply shifted attention in the direction of the gazer’s line of regard, or both. Samson and colleagues were aware of this potential problem and reported the results of a control experiment that ruled it out as an explanation of the consistency effect [4]. In one condition of this experiment, the gazing avatar was replaced by a similarly sized rectangle positioned in the same location in the centre of the virtual room. Samson et al. replicated the consistency effect with the avatars but not with the rectangles suggesting that whatever causes the effect, it is something to do with the directional information provided by the avatar and not a consequence of the arrangement of the target discs in the room. While the disc arrangement cannot be categorically ruled out as an explanation for the consistency effects observed in Experiments 1 and 2, the similarity with Samson et al.’s methodology suggests that it is not a strong candidate.

While the results of the present experiments suggest that the consistency effect is unlikely to be caused by spontaneous perspective taking, they clearly do not rule out the possibility that this kind of automatic mentalizing might occur in other tasks. One such example was recently reported by Surtees and colleagues [29]. On each trial in their study participants were asked to make a judgement about a number presented on a computer screen lying horizontally, embedded in a desk in front of them. In one condition judgments were made by the participant alone, while in another they were made in the presence of another individual who sat opposite them but who could also see the screen. The data of interest came from trials where the number to be judged by the participant looked the same or different from the perspective of the other individual (e.g., the numbers “8” and “6,” respectively). Judgments were made faster when perspectives were consistent (e.g. the number “8” which appears the same viewed upright or upside down) than inconsistent (e.g. a number “6” that appears as a “9” when viewed upside down). This appears to be an instance of level-2 perspective taking-understanding that a given object might appear differently to different viewers-and therefore does not lend itself to a simple gaze-cued orienting explanation: targets in both consistent and inconsistent conditions would always have been cued by the other individual’s gaze.

The Surtees et al. [29] study is also relevant here because, like Experiment 2, it involved participants completing the task while sitting face-to-face with another individual. However, merely seeing another “live” gazing agent was not sufficient to engage perspective taking. In follow-up experiments Surtees et al. showed that the consistency effect was only obtained when the second individual had already actively participated in a task that also involved the number targets. In this respect, the effect was not spontaneous but required the appropriate set of motivational circumstances to occur—those where the target stimuli were useful to both the participant and the other individual. This raises the possibility that the dot-perspective task might yield genuine spontaneous perspective taking if participants believed that the target dots were actually relevant for some task in which the gazing agent was either currently involved, or recently involved.

One possible limitation of the studies described here is that there was no control over attributions that participants might have made about the role of the gazer in the task. The same point might be levelled at any dot-perspective task in the literature but may be particularly pertinent in versions where participants are only required to make self-perspective judgements, as in Experiments 1 and 2 reported here and where they may be faced with a socially co-present gazer, as in Experiment 2. In Experiment 2, participants performed the task while sitting in front of a gazer whose behaviour was entirely unrelated to this task. Participants may nevertheless have made attributions about the gazer’s likely contribution to the experiment–whether or not they were acting collaboratively, for example. Duran et al. [30] showed that this kind of attribution influenced whether people were more or less likely to adopt an egocentric (i.e., self-perspective) or other-centric (i.e., other-perspective) frame of reference in a communicative spatial instruction task. This task was an analogue of the situation where two people are standing opposite one another on either side of a table. On the table between them are two folders, lying side by side so that one is to the left and one to the right of each person. If one of these people asks the other to “pass me the one on the left”, the recipient of the request has to make a decision about whose frame of reference “left” refers to: the requester’s or the recipient’s. In other words, the recipient must decide whether to assume their own perspective, or that of the person making the request. In the experimental version of this scenario, when participants were persuaded that the person making the request could not contribute to mutual understanding (e.g., because he or she was not in a position to judge the participant’s perspective), then participants were more likely to respond in a manner suggesting they were viewing the task from their partners’ perspectives. Conversely, when participants thought that their partners were taking full responsibility for working towards mutual understanding in the task, they (the participants) were more likely to adopt an egocentric frame of reference.

Quite how participants construed the gazer in Experiment 2 is not clear–a useful suggestion for future work would be to include measures to tap such attributions–but given the nature of the task it seems more likely that they would have considered his behaviour to be neutral rather than collaborative. According to Duran et al.’s [30] findings this ought to have stimulated a greater likelihood of perspective taking; however, as we have seen, the data do not support this interpretation. On the other hand, participants may have simply thought of the gazer as an irrelevant bystander–someone whose behaviour was entirely divorced from the experimental task. In this case, the data reported here do not really speak to the predictions arising from the theoretical framework used by Duran et al. [30].

Indeed, there are many differences between the present experiments and Duran et al.’s [30] work. In particular, the task used by Duran et al. seems likely to tap the slower, cognitively demanding system for reasoning about beliefs and desires proposed by Apperly and colleagues [2,3]. This system should indeed be capable of taking input from systems delivering attributional information about another individual; however, it seems unlikely that the fast, implicit mentalizing system posited to deliver spontaneous perspective taking would be so influenced. Nevertheless, future work might be aimed at assessing whether such attributions might play a role in stimulating spontaneous perspective taking.

In summary, the experiments presented here offer a stringent test of the spontaneous mentalizing hypothesis using photorealistic stimuli and a novel face-to-face version of the dot-perspective task which participants completed in front of a co-present gazer. In neither case was there any evidence in support of the claim that others’ perspectives are automatically computed, at least in the dot-perspective task. The likelihood is that the positive results of studies using this paradigm are caused by gaze-cued orienting of attention, which is unlikely to involve mental state representations.

## Figures and Tables

**Table 1:** Percentage of errors and standard deviations (in parentheses) in each condition of Experiments 1 and 2.

Experiment	Seeing		Non-Seeing	
	Consistent	Inconsistent	Consistent	Inconsistent
Experiment 1	3.9 (5.0)	3.7 (4.6)	6.9 (6.9)	5.0 (6.0)
Experiment 2	6.6 (8.8)	6.7 (8.4)	7.3 (7.4)	5.7 (7.3)
